# Tracking facility-based perinatal deaths in Tanzania: Results from an indicator validation assessment

**DOI:** 10.1371/journal.pone.0201238

**Published:** 2018-07-27

**Authors:** Marya Plotkin, Dunstan Bishanga, Hussein Kidanto, Mary Carol Jennings, Jim Ricca, Amasha Mwanamsangu, Gaudiosa Tibaijuka, Ruth Lemwayi, Benny Ngereza, Mary Drake, Jeremie Zougrana, Neena Khadka, James A. Litch, Barbara Rawlins

**Affiliations:** 1 Jhpiego Baltimore, Baltimore, MD, United States of America; 2 Jhpiego Tanzania, Dar es Salaam, Tanzania; 3 Ministry of Health, Community Development, Gender, Elderly and Children, Dar es Salaam, Tanzania; 4 Johns Hopkins Bloomberg School of Public Health, Department of International Health, Baltimore, MD, United States of America; 5 Save the Children, Washington, DC, United States of America; 6 Global Alliance to Prevent Prematurity and Stillbirth, Lynnwood, WA, United States of America; Johns Hopkins School of Public Health, UNITED STATES

## Abstract

**Background:**

Globally, an estimated 2.7 million babies die in the neonatal period annually, and of these, about 0.7 million die from intrapartum-related events. In Tanzania 51,000 newborn deaths and 43,000 stillbirths occur every year. Approximately two-thirds of these deaths could be potentially prevented with improvements in intrapartum and neonatal care. Routine measurement of fetal intrapartum deaths and newborn deaths that occur in health facilities can help to evaluate efforts to improve the quality of intrapartum care to save lives. However, few examples exist of indicators on perinatal mortality in the facility setting that are readily available through health management information systems (HMIS).

**Methods:**

From November 2016 to April 2017, health providers at 10 government health facilities in Kagera region, Tanzania, underwent refresher training on perinatal death classification and training on the use of handheld Doppler devices to assess fetal heart rate upon admission to maternity services. Doppler devices were provided to maternity services at the study facilities. We assessed the validity of an indicator to measure facility-based pre-discharge perinatal mortality by comparing perinatal outcomes extracted from the HMIS maternity registers to a gold standard perinatal death audit.

**Results:**

Sensitivity and specificity of the HMIS neonatal outcomes to predict gold standard audit outcomes were both over 98% based on analysis of 128 HMIS–gold standard audit pairs. After this validation, we calculated facility perinatal mortality indicator from HMIS data using fresh stillbirths and pre-discharge newborn death as the numerator and women admitted in labor with positive fetal heart tones as the denominator. Further emphasizing the validity of the indicator, FPM values aligned with expected mortality by facility level, with lowest rates in health centers (range 0.3%– 0.5%), compared to district hospitals (1.5%– 2.9%) and the regional hospital (4.2%).

**Conclusion:**

This facility perinatal mortality indicator provides an important health outcome measure that facilities can use to monitor levels of perinatal deaths occurring in the facility and evaluate impact of quality of care improvement activities.

## Introduction

Globally, an estimated 2.7 million babies die in the neonatal period annually, and of these, approximately 0.7 million die from intrapartum-related events [1]. Almost all of these deaths (98%) occur in low- or middle-income countries (LMIC) [2]. Very early newborn deaths (i.e., deaths in the first 24 hours after birth) make up a substantial portion of the overall burden of newborn and child deaths. A recent study of six LMIC countries put the proportion of very early newborn death to all neonatal mortality at 46% across six countries studied [3].

Many perinatal deaths can be prevented, [4,5] especially in a health facility setting [6]. In a multi-country study, 45% of perinatal deaths that occurred in the facility setting were found to be potentially preventable [6]. An assessment of audited perinatal deaths in Muhimbili National Hospital in Tanzania found that suboptimal care factors were present in 80% of the audited perinatal deaths [7]. Another modeling estimate found that globally, as many as 1.3 million intrapartum deaths and a large proportion of early newborn death could be averted with improved intrapartum care [8]. In an era of increasing institutional birth rates (in Tanzania, the institutional delivery rate increased from 50% in 2010 to 63% in 2015) [9], a facility-specific perinatal mortality indicator is highly relevant for guiding initiatives to improve the quality of facility intrapartum and postnatal care.

Perinatal death is defined by WHO as deaths that occur within the period of 22 weeks of gestation up to seven days of life, and neonatal mortality is defined as death of a live born baby within 28 days of life [10]. Tanzania is a major contributor to the global burden of perinatal mortality with 51,000 neonatal and 43,000 stillbirths occurring every year [11,12]. Although reduction in under-five mortality in Tanzania has been impressive, the reduction in neonatal mortality has been much slower, and an estimated 40% of deaths in children under five in Tanzania occur among neonates [13]. Baqui et al. described Tanzania as having one of the highest proportion of neonatal deaths occurring in the first 24 hours (65.5%) in LMICs [3]. Experts have estimated that two-thirds of these deaths could be prevented with improved intrapartum and neonatal care, such as provision of emergency obstetric care and focused care for vulnerable newborns [14].

In 2009, the World Health Organization (WHO) recommended collecting data on intrapartum and very early neonatal death rates as part of a larger set of emergency obstetric care indicators [15], noting that it is “a very important indicator enabling health personnel to take the most appropriate measures to prevent such deaths. Where women receive good care during childbirth, intrapartum deaths represent less than 10% of stillbirths due to unexpected severe complications [16].” Tanzania’s Ministry of Health, Community Development, Gender, Elderly and Children (MOHCDGEC) also considers rates of fresh stillbirth and pre-discharge neonatal deaths to be an indicator of quality of health care provided during the neonatal period [17]. This has been further supported by WHO’s multi-country network, launched in 2017, called the Quality Equity Dignity network, which is working to improve maternal, newborn, and child health [18].

Although there is agreement that improved quality of intrapartum care is essential for preventing deaths, accurately measuring deaths that occur during the intrapartum and newborn periods remains challenging [4]. Currently available measures of perinatal mortality have noted problems, including data quality such as non-recording of stillbirth and early neonatal deaths, inappropriate or inaccurate classification of stillbirths and very early newborn deaths, and difficulty in obtaining accurate gestational age or birth weight.

Without accurate data on location, frequency and timing of perinatal deaths, health facilities will have difficulty prioritizing areas for improvement. Merely improving the measurement of intrapartum deaths will not result in improvements in quality of care, rather, having reliable measurements of mortality is important to inform quality improvement efforts. For example, changes in the numbers or rates of perinatal mortality can be compared before, during and after the introduction of an intervention, such as improving newborn resuscitation skills of providers. Impactful quality improvement programs will link to data and monitoring of health outcomes. The indicator described in this study was investigated for its potential as a sensitive tool for measuring impact, embedded in largely available data and recommended clinical processes in Tanzania.

This study tested a facility-based indicator designed to be calculated using routine health information. The numerator validated in this study consisted of two components. The first was facility intrapartum deaths, specifically deaths before birth for fetuses with fetal heart tones detected and documented in the records at admission to the facility. The measurement of present (positive) or absent (negative) fetal heart tones at the time of admission of a woman in active labor renders the categorization of stillbirths as macerated versus fresh less relevant because the timing of death is known with certainty to be antenatal (i.e., before admission of a woman in labor) or intrapartum. The second component of the numerator was newborns born alive but who died before discharge from the health facility (newborn death before discharge). The denominator used in FPM indicator is “women admitted with a fetal heart rate detected” rather than “all women giving birth in the facility.” This denominator is specific to the cohort of women giving birth at the facility whose fetal and/or newborn outcome are directly impacted by the care, and quality of care, provided.

Using HMIS data to track routine service delivery indicators is critical for sustainable and cost-effective analysis of data for timely decision-making in LMIC [19]. Longstanding household survey programs, such as Demographic and Health Surveys (DHS) and Multiple Indicator Cluster Surveys (MICS), only generate data every 2–5 years and do not utilize clinical information that is captured in health facility records. In addition to being continuously rather than periodically available, routine health information for monitoring services is less expensive than population-based surveys [19]. The Tanzanian government, along with multiple countries in sub-Saharan Africa, uses district health information system version 2 (DHIS2) as an electronic platform for storing and analyzing aggregate health service data. An indicator built into the DHIS2 that tracks facility-based perinatal mortality could be a powerful metric to guide and monitor efforts to improve quality of intrapartum and postnatal care. Tanzania’s DHIS2 system does not currently have such an indicator.

This study aimed to validate a facility perinatal mortality (FPM) indicator by comparing health facility register data to gold-standard audit data. After validating the specificity of HMIS data using the audits, we used the study facilities’ HMIS data to calculate an indicator of facility-based intrapartum fetal death and pre-discharge neonatal mortality, or FPM. We present new information on the validity of HMIS data on perinatal mortality in Tanzanian health facilities, and show an example of how to calculate this FPM indicator at the facility level. This new metric enables tracking of FPM, which can inform efforts to improve intrapartum care in LMIC facilities.

## Methods

### Study aims

This study had two primary aims. First, to determine the sensitivity and specificity of perinatal death outcomes recorded in the HMIS as compared to a gold-standard audit. Second, to use this validated HMIS-recorded perinatal death outcome information to calculate an indicator for facility based perinatal mortality.

### Study design and outcome measure

This quantitative study tested the validity of an indicator by comparing data from MOHCDGEC HMIS maternity register data to perinatal death audits. The study had a non-randomized design that included all health facilities in the region meeting a high volume delivery cutoff. Within these health facilities, all perinatal deaths that were written in the HMIS register during the six month study period were considered eligible for audit. The outcome measure associated with the audits was the sensitivity and specificity of the neonatal outcome—macerated stillbirth, fresh stillbirth and newborn death—recorded in the register, compared to the perinatal death outcome assigned from the associated audit. In other words, sensitivity and specificity were calculated to assess the degree to which the register was able to predict “true” causes of perinatal death according to the audit. Macerated stillbirths were included in the sensitivity and specificity calculations because of the demonstrated potential for misclassification of macerated and fresh stillbirths.

The second aim of the study, after validating the HMIS data through the audits, was to calculate the FPM indicator for each facility, drawn from data extracted from the HMIS. This aim was designed to illustrate the application of the indicator to facilities and to national and subnational authorities. One key difference between the audits and the indicator calculation was that macerated stillbirths were included in the register/audit comparisons but not in the FPM indicator calculation. This is further described below.

### Study sites

Kagera region in northwest Tanzania was selected for the study location because at the study baseline, it had the highest infant mortality rate in Tanzania (62 deaths per 1,000 live births, compared to the national rate of 46 deaths per 1000) [15]. Health facilities in Kagera region were considered eligible based on their involvement in the USAID Maternal and Child Survival Program (MCSP), which supports the quality of maternal and newborn services. Facilities were included if they had at least 365 deliveries per year, were staffed by at least five skilled birth attendants, and were conducting perinatal death reviews, as stipulated by policy guidance from the MOHCDGEC in 2016 [10]. Facility delivery volume in 2015 (when the potential study site assessment was conducted) ranged from 385 to 4,388.

### Audits

Perinatal deaths that occurred in the study health facilities are, by protocol, recorded in the maternity register, which is part of the national HMIS. The starting point for the audits described in this study was a perinatal death that was recorded in the maternity register. Upon identifying a perinatal death in the maternity register, study staff followed up with the facility administrators to inquire when the audit would be scheduled and helped to schedule the audit if necessary.

According to MOHCDGEC standards, all perinatal and maternal deaths that occur associated with facility care should be audited [17]. Among the study audits, there was no case where there was both a maternal and perinatal death (i.e., only perinatal deaths were audited). The audits presented in this study were conducted as the facility’s own audit activities, with a study staff member present in the audit or drawn from clinical records of the audit. Audits were thus conducted following Tanzania’s Maternal and Perinatal Death Surveillance and Response (MPDSR) guidelines [10].

The comparison of HMIS-registered perinatal deaths with audits were conducted for perinatal deaths that occurred in the facilities from November 2016 to April 2017. Macerated stillbirths were included in the validation exercise (but not in the calculation of the FPM indicator) as they can commonly be misclassified as fresh stillbirth or newborn death and thus might affect the quantitative validation of the outcomes. In 81% of the audits (n = 104), study staff were present at the audit, took notes on the audit study form, and immediately after the audit used the facility’s MPDSR form to complete the study audit form. In 19% of the audits described (n = 24), study staff did not attend the audits, and rather used the facility staff’s records of the audit to complete the study form.

### Register data

Unlike the audits, in which perinatal death outcomes were assessed at individual level, the FPM indicator was designed to be calculated using aggregate data from the HMIS. To obtain all necessary information for calculation of the FPM indicator, study staff modified the HMIS maternity ward register (called MTUHA Book 12) of the study facilities. Two new columns were added manually: device used for fetal heart rate (FHR) assessment (specifying Pinard stethoscope or Doppler) and FHR in beats per minute (BPM) recorded on admission. Neither of these data elements are currently captured in the Tanzanian HMIS in a form reportable into DHIS2.

At the end of each month, a trained research assistant extracted this information from the maternity register using a study data collection form. This information was obtained for each full month that the study intervention period was conducted. The aggregated data were used to calculate the FPM rate for each facility.

### Maternity register data

The facility staff entered data on FHR and device used to assess FHR, and study staff regularly checked these data during the study. The register also collected information on birth outcome that was used for both the audit validation exercise as well as the FPM indicator calculation. To keep the indicator calculation feasible, the FPM indicator did not include gestational age (not included in the register) or a birthweight cutoff. Multiples were counted as multiple newborns. Data from the maternity register for the 6-month study period were extracted into the study’s aggregate data extraction forms monthly.

### Sampling and audit eligibility

For the HMIS classification (the “test” condition) to accurately detect newborn death, macerated stillbirth or fresh stillbirth on audit (the “gold standard” condition) with a minimum of 85% accuracy (i.e., sensitivity), a potential effect size of any short-term intervention +/- 7%, and within a pre-established alpha of +/- 0.05% significance, a required sample size of 106 “pairs” of HMIS registered and audited perinatal deaths was used.

Existing sources of data available in 2015 and co-investigator expert consensus were used to arrive at a estimate of baseline facility perinatal mortality of 12 per 1000 total deliveries. We used the following information and assumptions: census data showed Tanzanian neonatal mortality was 26/1000 births [15], roughly 50% of Tanzanian deliveries occurred in facilities during the 2015 calendar year [13], and approximately 50% of perinatal mortality could be expected to be documented in facilities. Thus, concluding that 12/1,000 deliveries was a conservative estimate of less than 50% of the perinatal mortality, we applied this estimated rate to the power calculation to yield an estimated 8,830 births needed to obtain the required 106 “pairs.” Assuming that baseline delivery figures would remain steady throughout the study period, the 10 study facilities included were expected to record approximately 9,050 births over the 6 months of the study, which allowed room for practice-based error (e.g., some newborns would be delivered without having FHR measured and recorded).

### Quality assurance of audits

All perinatal death audits included in the study were assessed for quality (clarity of recording, completeness and soundness of conclusions) by two medical doctors, one based in Kagera (BN) and one based in Baltimore (MCJ), before inclusion in the study analysis. A total of 15 audits were excluded because of missing information.

### Training and supervision

Research assistants for the study were nurses or nurse-midwives with related training and/or experience in research. Research assistants attended an initial four-day training on research ethics, study procedures, including best practices for attending audits, extracting data from the HMIS, using the hand-held Doppler device, and observing and interviewing health care providers. This training also included an orientation to data collection tools and standard operating procedures. A regional study coordinator conducted on-site and remote supervision of research assistants during the data collection period.

Health care providers in the maternity ward were trained to use the Doppler for admission to maternity services, appropriate case management practices when an abnormal FHR was detected and enter the information into the facility maternity register. The provider training took half a day and occurred on-site in the study facilities. Provider competency in using the Doppler and register was assessed using a pre-tested, standardized training protocol and observed structured clinical examination (OSCE) for skills in admission to labor and FHR measurement and knowledge assessment. We invited all of a facility’s health care providers with admitting privileges to participate in the study training. Following training, each study health facility was equipped with two hand-held Doppler devices, to be used during the admission to maternity services to assess fetal heart tones. Other uses of these Doppler devices in the maternity ward, for instance intermittent monitoring throughout labor, may have occurred but were not the subject of this study and thus no information was collected on broader clinical use of the Doppler.

### Data collection

Data were collected between November 2016 and April 2017. One research assistant was assigned to two health facilities and supervised completion of the register data, attended perinatal death audits, encouraged facility staff to use the Doppler device, and extracted data from the maternity register. The study staff also worked to bring perinatal deaths to the attention of facility administrators so that the MPDSR audits could be scheduled.

Study staff regularly assessed the working condition of the Doppler at the facilities, addressed any technical problems using the Doppler, and encouraged facility staff to use Dopplers for every admission to maternity services. Over the course of the study, a few staff who had not been present during study training worked in admissions. Some staff preferred using the Pinard stethoscope rather than the newly provided Doppler to measure FHR.

### Definitions

This study tested the feasibility and validity of a user-friendly facility measure of pre-discharge perinatal mortality (occurring within the health facility). Given the facility-based nature of the study, our study definitions of perinatal death outcomes differ slightly from both the Tanzanian definitions and those described by WHO in their 2016 guidance [20]. The definitions we used in the study are most similar to the 2009 WHO-proposed indicator, which is called “intrapartum and very early neonatal death rate.” The definition of WHO’s indicator is “intrapartum and newborn death within the first 24 hours of life” (numerator) and “all women giving birth in the facility in the same period (denominator) [8].” The major difference between the 2009 WHO indicator and the one tested in the study is that we have used “death before discharge” in the numerator rather than “death within 24 hours” and “women admitted with a FHR detected” rather than all women giving birth in the facility for the denominator. In the WHO indicator, it is recommended that neonates weighing less than 2,500 grams be excluded from the numerator if data allow. Another indicator described by Measure Evaluation was also called intrapartum and very early newborn death rate, but excluded newborns weighing 2,500 grams or less [16]. The FPM indicator we calculated did not have a birthweight limit and did not consider gestational age. This was due to feasibility because we wanted health care providers or administrators to easily calculate the indicator. The study definitions used, compared to WHO and Tanzanian definitions, are summarized in Table 1.

**Table 1:** Study definitions compared to national and international definitions.

**Study terminology and definition**	**Tanzania terminology and definition**	**WHO terminology and definition**
**Intrapartum and Very Early Newborn Deaths**		
Facility intrapartum and pre-discharge neonatal deaths: Intrapartum stillbirths and newborn deaths that occur before discharge from facility (excluding macerated stillbirth)	Perinatal death (no exact equivalent definition): Death of a fetus from 28 weeks of gestation to 7 completed days of life including stillbirths (macerated and fresh) ^a^	Intrapartum and very early newborn death rate: Proportion of births that result in newborn death or intrapartum death (fresh stillbirth)^b^
**Facility Perinatal Mortality (FPM)**		
Perinatal death that occurs in the facility setting (woman admitted with a FHR detected, discharged with neonate deceased)	*No equivalent definition*	*No equivalent definition*
**Very Early Newborn Death**	**Early Neonatal Death**	**Very Early Newborn Death**
Perinatal death recorded in maternity register as live birth for which a perinatal death audit was conducted OR Perinatal death classified by perinatal death audit as live birth, dead at discharge from facility	Death within 24 hours of delivery	Death within 24 hours of delivery
**Fresh stillbirth**		
Perinatal death where woman was admitted with a FHR detected and baby was born dead OR Perinatal death classified by perinatal death audit as fresh stillbirth	Stillbirth occurring intrapartum, with skin still intact, implying death less than 12 hours before delivery, with added criteria of weight more than 1000 g, and being born after more than 28 weeks’ gestation, and excluding those with severe lethal congenital abnormalities ^a^	Stillbirth occurring after onset of labor and before birth (intrapartum) with a fresh or non-macerated appearance of the skin.

^a^ MOHCDGEC, Tanzania, 2015, Maternal and Perinatal Death Surveillance and Response.

^b^WHO. 2009. Monitoring emergency obstetric care: a handbook.

#### HMIS maternity register variables

We used the HMIS maternity ward register (MTUHA Book 12) variable to establish the register-recorded perinatal outcome. Numbers of stillbirths were taken from the register from a section in the register called obstetric problems, birth outcomes and condition of the mother and newborn at discharge (*Matatizo ya mimba*, *Matokeo ya Uzazi na Hali ya Mama na Mtoto*) and the column listing fresh stillbirth/macerated stillbirth. Condition of mother and child at discharge from the facility (*Hali ya Mama na Mtoto wakati ya kuruhusiwa kutoka kituo cha huduma za afya*), which tracked relevant data in three columns (child alive/dead; date of discharge or death; and cause of death), was utilized to identify cases of very early newborn death.

#### Audit

The study audit form, based on Tanzania’s national MPDSR form with the addition of a limited number of variables from WHO’s perinatal death audit form, and the presence of absence of fetal heart tones at admission, was used to collect and analyze data for the “gold standard."

#### Data management

Study forms (audit forms and monthly aggregated data extraction forms) were collected on paper and subsequently entered by a data clerk into a password protected, web-based CommCare database. The database contained several range and value checks for quality and had drop-down menus with standardized values.

#### Analysis

For the analysis of register data compared to audit data (indicator validation), we calculated the sensitivity and specificity of the register to correctly detect audit (gold standard) perinatal outcomes. We conducted descriptive statistics on the data extracted from HMIS registers. Data were exported from CommCare and analyzed in SPSS v24 (IBM, Austin Texas).

### Ethical oversight

This study was submitted to the Johns Hopkins Bloomberg School of Public Health Institutional Review Board (IRB) and was determined to be exempt (JHU IRB #00007059). The study was approved by the Tanzania National Institute of Medical Research (NIMR) IRB (NIMR/HQ/R.8a/vol.IX/2219).

## Results

### Service provision and perinatal outcomes in the study

A total of 9,687 women were admitted to the maternity services in the study facilities from November 2016 to April 2017 (Table 2). Of these, 9,411 (97%) had FHR assessed upon admission. A total of 326 perinatal deaths were recorded, including 76 fresh stillbirths, 99 macerated stillbirths and 151 newborn death. The crude rate of perinatal deaths (including macerated stillbirths, with all admissions as a denominator, not adjusted for multiples) was 3% (326 deaths of 9,687 admissions). The perinatal mortality rate was 34 per 1,000 live births.

**Table 2:** Overview of maternity services and outcomes in facility perinatal mortality study facilities, November 2016 –April 2017.

	Total	Regional Hospital	District Hospitals						Health Centers			
		Regional Hospital A	District Hospital A	District Hospital B	District Hospital C	District Hospital D	District Hospital E	District Hospital F		Health Center A	Health Center B	Health Center C
**Service provision**												
Admissions	**9,687**	2,383	740	825	662	339	1,240	1,647		933	201	717
Vaginal deliveries	**7,888**	1,999	655	622	483	223	910	1,346		841	185	624
Cesarean deliveries	**1,864**	429	128	214	197	116	357	322		0	0	101
**Perinatal outcomes**												
Newborn death	**151**	71	15	16	4	6	8	27		2	1	1
Fresh stillbirths	**76**	29	4	2	6	4	22	7		1	0	1
Macerated stillbirths	**99**	36	2	7	6	6	8	26		1	1	6
Total stillbirths	**175**	**65**	**6**	**9**	**12**	**10**	**30**	**33**		**2**	**1**	**7**
Total perinatal mortality*	**326**	**136**	**21**	**25**	**16**	**16**	**38**	**60**		**4**	**2**	**8**
**FHR Assessment**												
Women admitted, FHR assessed	**9,411**	2,379	740	662	661	339	1,189	1,594		932	198	717

*Total perinatal mortality = newborn deaths + total stillbirths

### HMIS and audit comparisons

A total of 128 perinatal deaths (macerated stillbirths, fresh stillbirths and newborn death) were examined as audits compared to HMIS-registered perinatal outcomes in the study facilities during the study period (Table 3). Audits were conducted at every facility in the study. The number conducted correlated to the number of perinatal deaths at that facility, meaning audits were not evenly distributed across facilities. The range was 2–32, and median of 6 audits conducted per facility. Although the audits included macerated stillbirths, these were not included in the FPM indicator calculation because a macerated stillbirth would have occurred before admission to the facility, and would have been unaffected by care received at the facility level. Macerated stillbirths were, however, included in the sensitivity and specificity calculations to address the commonly-observed potential for misclassification of macerated and fresh stillbirth. Of the 128 perinatal death audits, 104 were observed by research team members and 24 were retrospectively reviewed.

**Table 3:** Perinatal death audits in the FPM study.

Audits	
Newborn death (death before discharge)	44
Macerated stillbirth	41
Fresh stillbirth	43
**Total audits conducted**	**128**
Average number of days from death to audit (range)*	16.4 (SD 14.4; median 11.5; range 0–59)
Audits conducted with study staff present	104 (81.3%)
Audits included after rigorous retrospective review	24 (18.7%)

*Of 98 audits with adequate information.

Perinatal death outcomes from the maternity register were compared with perinatal death outcomes from the associated facility perinatal death audit (Table 4). In only one case, the outcome differed. In this case, a perinatal death listed as fresh stillbirth in the HMIS was determined in the audit to be a newborn death.

**Table 4:** Outcomes of the perinatal death audit compared to outcomes recorded in register.

		Audit Perinatal Death Classification			
		Newborn death	Fresh stillbirth	Macerated stillbirth	Total
**Register Perinatal Death Classification**	**Newborn death**	44	0	0	44
	**Fresh stillbirth**	1	45	0	46
	**Macerated stillbirth**	0	0	43	43
	**Total**	45	45	43	133

The sensitivity and specificity of HMIS register data to predict the gold standard audit outcomes was calculated. All outcomes (fresh stillbirth, macerated stillbirth, and newborn death) had high sensitivity and specificity values (Table 5). The sensitivity (probability of stillbirth or newborn death in the register given that it was classified as such in the audit) was 95.7%, 100% and 97.8% for fresh stillbirth, macerated stillbirth and newborn death, respectively. The specificity (probability of not stillbirth or not newborn death in the register given that it was classified as such in the audit) was 98.8%, 100% and 97.7%, for fresh stillbirth, macerated stillbirth and newborn death, respectively.

**Table 5:** Sensitivity and specificity of perinatal death outcomes in HMIS register to predict perinatal death outcomes of audit.

OUTCOME		Fresh Stillbirth		Macerated Stillbirth		Newborn Death	
		Prevalence	95% CI	Prevalence	95% CI	Prevalence	95% CI
Prevalence	Pr(A)	35%	(27% - 43.6%)	31%	(23% - 39.7%)	34%	(26–42.8%)
Sensitivity [probability of stillbirth or newborn death in register given it is classified so in the audit]	Pr (+|A)	95.7%	(85.2% - 99.5%)	100%	(91.4% - 100%)	97.8%	(88.2% - 99.9%)
Specificity [probability of not stillbirth or not newborn death in the register given it is classified so in the audit]	Pr (-|N)	98.8%	(93.7% - 100%)	100%	(96% - 100%)	97.7%	(91.9% - 99.7%)

### Facility perinatal mortality indicator

The proposed facility perinatal mortality indicator is designed to help facilities assess perinatal deaths that occur in the facility setting, using HMIS data. The indicator is defined as follows:

$$\frac{Fresh\;stillbirth+very\;early\;newborn\;death}{All\;admissions\;with\;FHR\;detected}$$

The FPM indicator by facility during the 6-month study period is presented in Table 6. FPM rates varied from 0.3% to 4.2% (Table 6). Regional and district hospitals had consistently higher FPM indicator rates compared to the health centers.

**Table 6:** Facility perinatal mortality indicator by facility, November 2016 –April 2017.

Facility	Fresh stillbirths	Newborn deaths	Women admitted with FHR assessed	FPM Indicator calculation	FPM %
**Regional Hospital**					
Regional Hospital A	29	71	2379	$\frac{29+71}{2379}=0.042$	4.2%
**District Hospitals**					
District Hospital A	4	15	740	$\frac{4+15}{740}=0.026$	2.6%
District Hospital B	2	16	662	$\frac{2+16}{662}=0.027$	2.7%
District Hospital C	6	4	661	$\frac{6+4}{661}=0.015$	1.5%
District Hospital D	4	6	339	$\frac{4+6}{339}=0.029$	2.9%
District Hospital E	22	8	1189	$\frac{22+8}{1189}=0.025$	2.5%
District Hospital F	7	27	1594	$\frac{7+27}{1594}=0.021$	2.1%
**Health Centers**					
Health Center A	1	2	932	$\frac{1+2}{932}=0.003$	0.3%
Health Center B	0	1	198	$\frac{0+1}{198}=0.005$	0.5%
Health Center C	1	1	717	$\frac{1+1}{717}=0.003$	0.3%
**Total**	**76**	**151**	**9,411**	$\frac{76+151}{9411}=0.024$	**2.4%**

**Fig 1:**
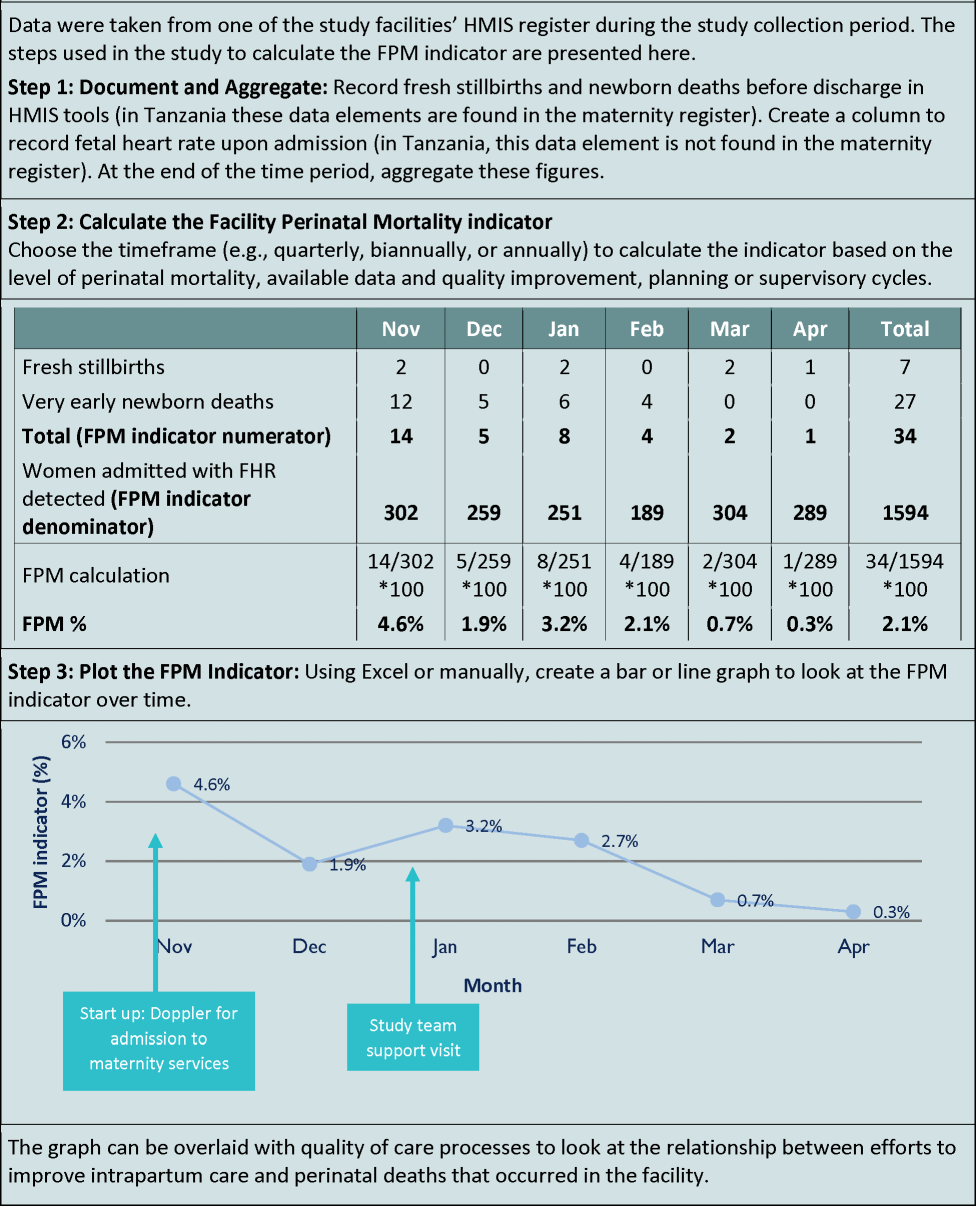
Case study calculating facility perinatal mortality indicator.

Fig 1 shows an example of applying the indicator to track facility-based intrapartum stillbirth and newborn death, using data from one of the study health facilities.

## Discussion

This study was designed to provide a practical method for measuring perinatal mortality occurring within health facilities, which can be calculated using HMIS data. Tracking rates of facility-based perinatal mortality can inform quality improvement initiatives at facilities, and can guide management decisions, for example, helping district authorities prioritize which facilities get additional support to improve intrapartum care. The calculation is most useful when stillbirth outcome classification is highly accurate. To establish the indicator validity, we assessed the sensitivity and specificity of HMIS data and found both of these to be high.

The high level of agreement between perinatal death outcomes in the HMIS register and the audits was encouraging because it suggested that the FPM indicator calculation is relatively accurate and thus potentially useful for measuring facility-based perinatal death. However, high accuracy of classification of perinatal death outcomes has not been reported consistently across the literature. In Ghana, a study assessing the effectiveness of visual classification of perinatal death found that one-third of fresh stillbirths were reported as macerated, and half of the macerated stillbirths were described as fresh, causing the authors to question whether appearance (the method of classification generally used in Tanzania) is an accurate proxy for pre-partum versus intrapartum death [21]. In Tanzania, a similarly high level of misclassification of perinatal death became evident when an intervention to improve newborn resuscitation unexpectedly resulted in a reduction in fresh stillbirth, indicating that health care providers had been mistakenly classifying newborn deaths as fresh stillbirths [20]–perhaps from bias, perhaps due to error. The benefit of the proposed indicator is that errors in classification between fresh stillbirth and newborn death will not change the value as both are captured in the numerator. Indeed, we note that in our findings, the one perinatal death that was misclassified in the register was a fresh stillbirth that was determined by audit to be newborn death. This misclassification would not have affected the FPM indicator values upon calculation.

The MPDSR was established in Tanzania in 2006 to promote a culture of quality in the health facility setting, where “every maternal (and perinatal) death counts and needs to be investigated [17].” The Ministry recognized in the MPDSR guidance that misclassification of perinatal deaths is common [17]. In 2016, WHO issued new guidance on perinatal death audits [22], along with clarification about classification of perinatal death [23]–both of these are useful resources for planning training, which may be potentially incorporated into ongoing pre- and in-service education. Tanzania’s 2015 MPDSR guidelines do not include an example for calculating facility level rates of perinatal mortality [17]. Joining training for misclassification with training on how to calculate the FPM indicator may be useful for overall quality improvement processes in facilities. The training and competency assessment for health care providers associated with this study, which covered use of the Doppler and recording and classification of perinatal deaths, was conducted on-site and took 3–4 hours.

Existing measures to track perinatal mortality are not very specific. For example, facilities can look at a crude rate of perinatal deaths out of all admissions; in the case of our study facilities this was 3% (326 deaths of 9,687 admissions). A study at Tanzania’s Kilimanjaro Christian Medical Center hospital found a rate of 3.5% [24]. This rate, being out of all admissions, may include women admitted with a macerated stillbirth. This dilutes the value of the measure to a facility administrator, as a fetal death that occurred before admission to the health facility has no relation to the quality of care at that facility. The proposed indicator allows facilities to have a specific assessment of the type of deaths that could potentially be averted with high quality of care.

WHO guidance cautions against calculating case fatality rate for time periods when the number of deaths is too small for a stable calculation [25]–this will hold true for the FPM indicator as well. It may be advisable for facilities with few perinatal deaths to calculate the indicator on a quarterly or even annual basis for increased stability of the indicator. More investigation is needed to refine the application of the indicator. Questions to answer include at which level levels of perinatal mortality is a stable rate produced and how frequently should the indicator be calculated, timing of calculation, the appropriate FPM rate at various levels of the referral chain, and expected timing and level of reductions of FPM given quality improvement. All of these questions will benefit from facilities carefully documenting their experiences as they apply the FPM indicator.

The use of HMIS data has many benefits, including that it is readily available in all public health facilities. However, there are drawback to HMIS data, the major one being completeness, as shown by an assessment in Kenya that documented real challenges to application of DHIS2 data for decision making based on incompleteness [19]. Calculation of this indicator to track facility-based perinatal mortality should include efforts to improve quality of HMIS data, as well as a realistic assessment of the quality and completeness of these data. This indicator has deliberately simple numerator components to make the indicator more accessible to health facility staff. Additionally, these will hopefully reduce necessary modifications to HMIS.

Calculating the FPM indicator requires consistent assessment and recording of FHR upon admission to maternity services. In our study’s procedures, FHR upon admission was verified using a hand-held Doppler device. However, the FPM indicator could be calculated with either a hand-held Doppler or a Pinard stethoscope. The diagnostic accuracy of hand-held Doppler compared to the Pinard stethoscope for measuring FHR has not yet been reported. Additionally, there is no information in the literature about the extent to which Doppler versus Pinard stethoscope is used in Tanzania. Our experience with both devices leads us to recommend Doppler for assessment of FHR upon admission because of reliability (the Doppler reads out the FHR digitally) and end-user experience (the FHR is audible to the mother and provider). We found the introduction of Doppler to be feasible requiring a 3–4 hour training of health care providers, highly acceptable for routine use by health care providers, and the Doppler device to be robust during daily use.

The indicator must be considered in context. The FPM indicator tested in this study is intended to support analysis of a single facility’s trends in perinatal mortality over time, rather than comparing different facilities. This is because differences in levels of care and the client base may significantly contribute to overall levels of mortality within the facility. For example, a facility that has a geographically remote catchment area may have more women who have experienced significant delays in reaching the facility, poorer fetal condition upon admission, and higher perinatal mortality at that facility compared to another. Our study was not sampled to detect differences in FPM rate at different levels of health facility. However, differences emerged, with hospitals having higher levels compared to health centers, and the regional hospital having the highest level. This is likely due to higher-level facilities handling more high-risk cases and obstetric emergencies. Because of this lack of comparability across facilities, this indicator is not suited to serve as a national or an international standardized measure of perinatal mortality. Although the FPM indicator as tested in this study was primarily intended to support analysis of a single facility’s trends in perinatal mortality over time, in the longer term, this indicator may have strong utility at the sub-national level, and may be useful to incorporate into DHIS2.

This study methodology—using perinatal death audit as a tool to validate perinatal deaths recorded in the HMIS—is particularly timely given a recent push to increase the use of MPDSR to improve quality of maternal and newborn health care in Tanzania [17]. All of the study facilities received support from the USAID Maternal and Child Survival Program to improve perinatal death audits—both for the study as well as ongoing programmatic support. Facility-level perinatal death audits are not easy for health facilities to perform, as highlighted by a recent editorial on the challenges of the rollout of WHO’s MPDSR guidance [26]. A qualitative study in Tanzania documented related challenges, particularly pertaining to perinatal death reviews [27]. The challenges around conducting perinatal death audits were apparent in our study. Study fieldwork notes indicated that audits were challenging for health facilities to plan, schedule and conduct, mainly due to shortages of health facility staff. Not all perinatal deaths that occurred in the study health facilities during the study period were audited, although they should have been. The mean time for audits to be conducted was 16 days after the death occurred, which is over twice the time frame recommended by the Ministry [17]. The study thus confirmed reported challenges, but also provided an example of how audits can be improved, and can produce useful information for improving quality of care.

This study had some limitations. By using facility-run perinatal death audits, the gold standard audit was practically the highest available standard that could be used in the study setting. However, these facilities had all had training and support in conducting MPDSR audits, and study staff were present in most audits and had a formative rather than observational role. Research staff were unable to attend all audits that occurred in the study health facilities during the study period. The roughly 20% of audits that were included in the study although study staff were not present at the audit were only included if they were legible, fully documented, complete, and came to clinically sound conclusions. All audits included in the study were checked by two medical doctors for quality: audits deemed to be of poor quality were excluded.

Low birth weight and preterm infants have higher mortality. By not including gestational age or a birthweight, we acknowledge that the tested FPM indicator is less sensitive to potentially preventable deaths, as preventing these deaths may either be harder or dependent on the sophistication of care at the facility. In facilities where it is feasible to stratify neonates by birthweight or gestational age, this is recommended. Future studies may wish to investigate causation between intervention and reductions in facility-based mortality, as well as examining time frames for expected changes based on data-driven assumptions about the intervention and environment.

This study audited all perinatal deaths reported in the study facilities in the study period, including macerated stillbirth, fresh stillbirth and very early newborn deaths. However, future studies may wish to focus solely on fresh stillbirth and newborn deaths because these have a more direct bearing on quality of intrapartum care.

## Conclusion

This study found a high level of sensitivity and specificity of perinatal deaths recorded in the HMIS registers in the study facilities compared to gold standard audits. The high sensitivity and specificity allowed for our conclusion that calculation of the facility perinatal mortality indicator (fresh stillbirth and very early newborn death divided by all women admitted to maternity services with FHR detected and recorded) is a valid and meaningful measurement for facilities to calculate and track in relation to quality improvements. This indicator is an important new tool for facilities to use to monitor improvements in quality of intrapartum care and the resulting reduction in preventable facility-based perinatal mortality.
